# Premenstrual syndrome and its association with exposure to political violence, human insecurity, and well-being: a cross-sectional study among Palestinian adolescent refugees

**DOI:** 10.1186/s12978-025-02104-z

**Published:** 2025-11-27

**Authors:** Nao Wakabayashi, Mohammed B. A. Sarhan, Rika Fujiya, Daisuke Sugiyama, Rie Fuse, Weeam Hammoudeh, Rula Ghandour

**Affiliations:** 1 Graduate School of Health Management, Keio University, Fujisawa, Kanagawa Japan; 2 Faculty of Nursing and Medical Care, Keio University, Fujisawa, Kanagawa Japan; 3 Institute of Community and Public Health, Birzeit University, Birzeit, Palestine

**Keywords:** Premenstrual syndrome, Exposure to political violence, Human insecurity, Palestinian refugees, Refugee camps, Adolescents’ sexual reproductive health, Menstrual health, Well-being

## Abstract

**Background:**

Premenstrual syndrome (PMS) is a common menstruation-related condition among adolescent girls. Vulnerability to environmental and social factors such as living under war, exposure to political violence (EPV), and human insecurity significantly influence the health and well-being of adolescents more generally. However, research on the association between PMS and social determinants in conflict settings remains limited. This study aimed to identify the severity of PMS and its association with EPV, human insecurity, and well-being among adolescent girls in Palestine refugee camps in the West Bank.

**Methods:**

This cross-sectional study included 1,399 girls aged 15–18 years residing in 19 Palestinian refugee camps in the West Bank, occupied Palestinian territory. PMS severity was measured using a scale developed based on the literature, expert input, and the girls’ experiences, comprising two categories: “none to mild” and “moderate to severe.” EPV was assessed based on past experiences at individual, familial, collective, and cumulative levels. Multivariate analyses were conducted using five regression models with a primary focus on the relationship between PMS severity and EPV.

**Results:**

The prevalence of PMS with at least one symptom was 92.1%. PMS severity was positively associated with collective EPV (adjusted odds ratio [AOR], 1.5; 95% confidence interval [CI], 1.1–2.1), whereas individual and familial EPV were only significant when included separately in the model. Girls who experienced two or three types of cumulative EPV (AOR, 2.5; 95% CI, 1.6–3.7) were more likely to experience severe PMS. High levels of human insecurity (AOR, 1.3; 95% CI, 1.0–1.6) and depression-like symptoms (AOR, 1.9; 95% CI, 1.3–2.7) were significantly associated with PMS severity.

**Conclusions:**

The results demonstrate a significant association between PMS severity and EPV, human insecurity, and low levels of well-being. These findings suggest that prolonged occupation and unresolved conflict may adversely impact adolescent health and exacerbate PMS symptoms, highlighting the need to recognize PMS as a public health concern. In protracted conflict settings, integrating psychosocial support and menstrual health education into schools and community-based programs such as primary healthcare facilities may help adolescent girls manage PMS, menstruation-related symptoms, and associated stressors more effectively.

**Supplementary Information:**

The online version contains supplementary material available at 10.1186/s12978-025-02104-z.

## Abstract (plain language summary)

Premenstrual syndrome (PMS) is a common symptom among adolescent girls occurring before menstruation, causing physical and mental symptoms such as headaches and low mood. PMS is not only related to lifestyle but also to trauma and the surrounding environmental and social conditions. People in Palestine have been exposed to political violence caused by long-term occupation and war for several decades, including restrictions on movement, economic instability, and daily insecurity. Political violence includes violence against individuals, such as home invasions and humiliation by soldiers; against families, such as imprisonment, injury, and the loss of family members; and against collectives, such as witnessing or hearing about political violence. Herein, we investigated the relationship between PMS severity and exposure to political violence (EPV), sense of insecurity, and well-being. We conducted home visits with adolescent girls aged 15–18 years living in refugee camps in the West Bank of Palestine. The results showed that 92.1% of girls experienced PMS symptoms, with PMS severity associated with EPV, high levels of insecurity, and low level of well-being. Furthermore, the presence of more types of EPV was strongly associated with PMS severity. These findings demonstrate the potential impact of long-term occupation and conflict on adolescent health, highlighting the need to address PMS as an important adolescent health issue, beyond simply causing discomfort. Integrated psychosocial support and menstrual health education in community- and school-based settings may enable adolescent girls to enhance their coping skills and manage menstruation-related symptoms, including PMS.

**Supplementary Information:**

The online version contains supplementary material available at 10.1186/s12978-025-02104-z.

## Background

Menstrual health is defined as the complete physical, mental, and social well-being in relation to the menstrual cycle [1]. Achieving good menstrual health for all women and girls is a major public health issue and a basic human right [2]. Premenstrual syndrome (PMS) is a prevalent menstrual health problem that affects girls from adolescence into adulthood [3]. PMS refers to the emotional, behavioral, and physical symptoms that occur during the luteal phase of the menstrual cycle (i.e. after ovulation) and usually disappear once menstruation begins. These symptoms include mood swings, tiredness, breast tenderness, and irritability, and can be mild or severe [4]. However, the etiology of PMS remains unclear and is intricately intertwined with many factors, including hormonal changes, eating habits, smoking, alcohol intake, menstrual characteristics, psychological factors, and traumatic experiences, including domestic violence, sexual abuse, emotional neglect, war, and natural disasters [5–12].

Globally, the prevalence of PMS is high, reaching 48% among women and girls [13]. Most women who have PMS begin to experience symptoms in their late teens or early twenties, gradually worsening over time [14–16]. In adolescent girls, PMS can have a significant negative impact on academic performance, extracurricular activities, interpersonal relationships, self-esteem, well-being, and quality of life [17, 18]. More generally, adolescence is a critical transitional stage with many physical, psychological, emotional, and social changes, making adolescents vulnerable to social stressors and environmental degradation, including armed conflict and migration at the regional and national levels [19–21].

Exposure to political violence (EPV) is a form of physical and psychological trauma and one of the main factors threatening adolescent health and well-being [22]. Political violence is defined as violent conflict or state violence perpetrated by a large group of people using power or force to harm and achieve political goals [23]. EPV has been identified as a contributing factor to the disruption of health services, food shortages, infrastructure collapse, and economic constraints. These consequences can lead to malnutrition, increased prevalence of infectious diseases, high rates of unemployment and poverty, and mental health conditions, including depression and anxiety disorders [22, 24]. EPV is also linked to sexual and reproductive health, gender-based violence, and menstrual cycle disturbances [24–29]. However, few studies have been conducted to establish sufficient evidence of its effects on menstruation.

Human insecurity is also an important concept when addressing adolescent health, especially in wartime conditions. Human insecurity threatens individuals’ safety, well-being, health, and rights across areas such as access to food, water, surrounding environmental conditions, and economic and political stability. These include violent conflict, extreme poverty, natural disasters, and pandemics, and as well as other issues faced by local communities [30–32]. Research has shown that menstrual insecurity, wherein girls and women lack adequate resources and facilities for menstrual hygiene, often results from broader economic and social, or political instability [33–35].

Occupied Palestinian territory (oPt) has experienced chronic and multigenerational trauma due to prolonged and ongoing occupation, and widespread EPV since 1948 [36]. Palestine refugees, particularly those residing in refugee camps, face unique vulnerabilities that profoundly affect their health and well-being. They are usually more prone to EPV, such as armed invasions of refugee camps, settler violence [37, 38], physical barriers to accessing healthcare facilities, financial difficulties, and chronic stressors that might negatively influence their mental health [39–43]. These exposures contribute both directly and indirectly to heightened human insecurity, posing threats to survival and resulting in significant psychosocial consequences [44, 45]. These conditions underscore the need to address cumulative EPV and human insecurity as structural barriers to health in conflict-affected settings [46].

Despite growing attention to the health consequences of political violence, research on the relationship between EPV and premenstrual syndrome (PMS) remains scarce. While some studies have examined links between PMS and traumatic experiences such as abuse or post-traumatic stress disorder, few have specifically investigated PMS in relation to conflict-related or political violence [11, 47]. In oPt, empirical evidence on PMS is extremely limited. Existing studies among university students in the West Bank report high PMS prevalence rates of 70% to 100% [48, 49].

PMS is chronic and cyclical, impacting health through the interaction of biological, cultural, social, economic, and political factors [50]. Despite its impact on the daily lives of adolescent girls, PMS remains under-recognized in public health research. Given its gender-specific nature and sensitivity to psychosocial stressors, PMS may serve as a valuable indicator of chronic stress, particularly in conflict-affected settings. Focusing on PMS provides unique insights into adolescent girls’ experiences of chronic stress in conflict settings, and highlights the need for context-specific public health research and intervention.

This study aimed to identify the severity of PMS and its association with EPV, human insecurity, and well-being among adolescent girls in Palestine refugee camps in the West Bank. By exploring this crucial but overlooked intersection, this study seeks to fill a significant gap in the literature and contribute to the formulation of more effective health policies that are acutely aware of and responsive to the complex interplay between political instability and health in conflict settings.

## Methods

This study is part of a larger implementation research project that examines the overall health status and needs of adolescent girls living in Palestine refugee camps and consists of three main phases: qualitative, quantitative, and interventional [51–53]. To contribute to this objective, data from a cross-sectional household survey conducted between June and September 2019, targeting adolescent girls aged 15–18 years living in any of the 19 Palestine refugee camps in the West Bank was used for our analysis.

### Study setting

Palestine refugee camps in the West Bank host families who were forcibly displaced from their homes after the Nakba following the 1948 Arab–Israeli war, as well as those displaced during the 1967 Arab–Israeli War [54]. In the West Bank, as of the second quarter of 2024, the number of registered refugees with the United Nations Relief and Works Agency for Palestine Refugees in the Near East is 1,148,391, of whom 15.9% are between 10 and 19 years of age [55]. Although Palestine refugee camps were built temporarily, a lack of political solutions and deteriorating economic conditions have given way to long-term conflict, resulting in densely packed living conditions due to population growth and poor infrastructure [56, 57].

### Sampling and data collection

To ensure representativeness, data were collected from all 19 Palestine refugee camps in the West Bank. The sample size was calculated using a stratified random sample proportional to the population of the refugee camp [52]. The inclusion criteria for this study were girls aged 15–18 years residing in Palestine refugee camps in the West Bank and having experienced at least one menstrual cycle. Data were collected using a random-walk door-to-door approach by trained female field workers who lived in camps or were familiar with camp settings [58]. This study targeted all households in refugee camps, and recruited participants from households with adolescent girls who met the inclusion criteria. Consent was first obtained from the mother or caregiver and then from the adolescent girl in that household. If more than one girl in the same household met the inclusion criteria, all were invited to participate in the study. Each interview was conducted separately and privately to ensure confidentiality. The study tools, including the questionnaires, were originally developed in Arabic, which is the native language of both the respondents and the research team. Therefore, no translation or back-translation was required.

### Study variables

#### Outcome variable: Severity of premenstrual symptoms

PMS scale was developed specifically for this study. As there is no standardized tool to measure PMS, we relied on the literature for relevant symptoms and supported them with what we achieved from the qualitative study and expert opinion [51]. The items of the PMS scale were developed based on a literature review, expert input, and qualitative data reflecting the lived experiences of adolescent girls in oPt. Content validity was ensured through consultation with medical professionals familiar with the local context. The internal consistency was assessed using Cronbach’s alpha.

A survey consisting of 23 questions on somatic, psychological, and emotional symptoms was administered to the participants, including whether they had experienced PMS symptoms five days before menstruation. The items used a 6-point Likert scale (“not at all” = 0 to “severe” = 5), with a total final score expected within the range of 0–115.

We dichotomized PMS symptoms to clarify the severity of PMS and its association with EPV, human insecurity, and well-being, as this was considered useful for clarifying public health interventions. In a previous study in Palestine, nearly half of the participants had severe PMS [48]. Thus, we adopted the median of 26 as the cutoff value and dichotomized the scores into “none to mild” (score, 0–26) and “moderate to severe” (score, 27–115). We further divided the PMS symptoms into the following four categories using interquartile ranges: “none” (score, 0), “mild” (score, 1–26), “moderate” (score, 27–39), and “severe” (score, 40–115) and with two categories. The Cronbach’s alpha for the PMS was 0.89, indicating sufficiently high internal consistency.

#### Independent variables: Exposure to political violence, human insecurity, and general well-being

EPV was assessed using validated questions from a previous study on Palestinian adolescents [59]. The EPV questionnaire comprised three domains: individual, familial, and collective. The first category included 14 individual EPV experiences with Israeli soldiers (e.g., exposure to a house search, body search, or other interaction that resulted in feelings of humiliation). The second category consisted of three items asking whether family members were affected by political violence (e.g., experience with family members being imprisoned, shot, injured, or martyred). The final category comprised 13 collective EPV related to no direct harm to oneself or one’s family but seeing or hearing someone else being subjected to EPV (e.g., exposure to the sound of bombing, seeing someone shooting at you). In each domain, participants were classified into two groups: those with (score = 1) and without (score ≥ 0) EPV. The possible score ranges were 0–14, 0–3, and 0–13 for individual, familial, and collective EPV, respectively. The Cronbach’s alphas were 0.59 for individual EPV, 0.60 for familial EPV, and 0.80 for collective EPV. While the individual and familial EPV scales exhibited relatively low internal consistency, a threshold of 0.6 is generally considered acceptable [60]. Moreover, political violence is a multidimensional concept aimed at capturing diverse forms of violence; hence, high inter-item correlations are not necessarily expected [61]. EPV is a dichotomous variable that indicates whether an individual has experienced political violence in the past. Therefore, Cronbach's alpha was used as a reference for reliability rather than for measuring latent factors.

A composite variable, “cumulative exposure to political violence,” was created by aggregating individual, familial, and collective EPV items (score range: 0–3). Participants were classified into three categories according to the number of EPV domains to which they had been exposed: none (score = 0), one (score = 1), and two or more (score ≥ 2). Cronbach’s alpha was 0.79 for cumulative EPV.

Human insecurity was assessed using 12 items related to loss of income, home, and land, and feelings of insecurity about the present and future political situation. The scale was validated among adults in the Gaza Strip and adolescent Palestinians living in the Gaza Strip and West Bank [62]. Responses for each item ranged from “never” (score, 0) to “extremely” (score, 4), with raw total scores ranging from 0 to 48. The score was converted to 100 by dividing the score by 48 and multiplying by 100. The cutoff point of 50 was used, with 0–50 considered as “low to moderate level of human insecurity” and 51–100 considered as “high level of human insecurity.” The Cronbach’s alpha for the scale was 0.80.

Subjective psychological well-being was measured using the World Health Organization’s Five Well-Being Index. This scale has adequate validity as a screening tool for depression risk and has been successfully adapted to a wide range of research areas [63]. This index consisted of five questions indicating self-assessment of positive well-being with answer categories ranging from 0 to 5 (“at no time” = 0 to “all of the time” = 5). The total raw scores range from 0 to 25. To obtain a standardized score, the raw scores were multiplied by four to obtain a score ranging from 0 to 100. Well-being was classified into three categories: “depression-like symptoms” (score, 0–28) [64], “low level of well-being” (score, 29–50), and “good well-being” (score 51–100) [65]. Cronbach’s alpha was 0.89.

#### Other independent variables

Other independent variables included demographic and socioeconomic information, menstrual characteristics, and other health behaviors. Regarding demographic characteristics, girls were asked to disclose their age, location (close to urban or rural), economic status, marital status, parents’ educational level, and frequency of physical activity. Economic status was reported based on subjective perceptions.

Information regarding menstrual patterns was collected by directly asking the girls about their age at menarche, menstrual cycle length, menstrual duration (number of bleeding days), heaviness of bleeding, and time since menstruation [52]. Menstrual characteristic variables were classified according to the criteria of the American College of Obstetricians and Gynecologists (ACOG) criteria [66]. Bleeding severity was measured on a Likert scale using a proxy question (e.g., whether blood leaked from the girl’s clothing during the first two days of menstruation) [67–69], comprising “never,” “rarely to sometimes,” and “almost to always.”

### Statistical analysis

Data were analyzed using SPSS (version 28) (IBM Corp., Armonk, NY, USA). Non-responses were considered as missing data. Specifically, there were no responses to questions related to familial EPV, the menstrual cycle, human insecurity, or well-being. This might be because these questions reflected their distressing experiences, leading them to refrain from answering them. Furthermore, because menstruation is a sensitive topic, some participants may hesitate to answer the questions. Nine participants (0.6%) did not answer any of the questions and were excluded from statistical analysis. Given this small proportion, we did not explore the patterns or mechanisms of missing data.

Descriptive analyses were conducted to determine the participant characteristics. The prevalence of PMS severity is shown in four levels (“none,” “mild,” “moderate,” and “severe”) using quartile ranges and the types of PMS symptoms for all participants.

**Fig. 1:**
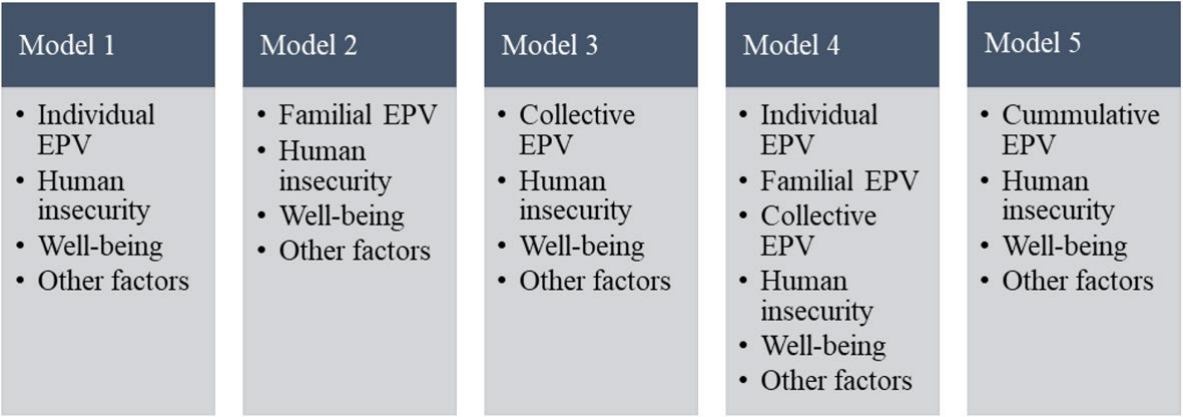
Regression models investigating the association between PMS severity (outcome) and EPV

Multiple binary logistic regression was performed using the dichotomized PMS variable as the outcome to examine the associations with EPV, controlling for all other variables. Five models were used to further examine the association between each type of EPV and PMS severity (Fig. 1). Models 1, 2, and 3 included individual, familial, and collective EPV, respectively; Model 4 introduced the three EPV scales into one model; and Model 5 included the cumulative EPV scale. All models controlled for demographic and socioeconomic information, menstrual characteristics, health behaviors, human insecurity, and well-being variables.

Although a formal prior power analysis was not conducted, the sample size was considered adequate, as the number of events (moderate to severe PMS) allowed for the inclusion of up to 67 independent variables in the model [70].

Sensitivity analysis was performed for different population targets to ensure the robustness of the results. In this study, although married girls were excluded from the sensitivity analysis because of their small proportion, similar results were obtained. The absence of multicollinearity in all the regression models was confirmed by conducting variance inflation factors and tolerance tests. All statistical tests were two-sided, and the significance level was set at *p* < 0.05. Adjusted odds ratios (AOR) with 95% confidence intervals (CI) were reported.

### Ethics approval

This study was approved by the Research Ethics Committee of Birzeit University, Palestine (approval no. 171114) in December 2017. Signed consent was obtained from the girls and their parents in each household. Data were collected anonymously to ensure confidentiality and privacy.

## Results

A total of 1399 girls aged 15–18 years from 19 Palestinian refugee camps in the West Bank participated in this study (Table 1). The response rate was 87% at the household level and 95% among adolescent girls. The average age was 16.9 ± 1.2 years. Most participants were single (98.0%), and only a small number of women were married (2.0%).

**Table 1:** Participant characteristics

Demographic and socioeconomic characteristics	n (%)
Age (*n* = 1399)	16.9 ± 1.2
15 years	404 (28.9)
16 years	324 (23.2)
17 years	374 (26.7)
18 years	297 (21.2)
Marital status (*n* = 1399)	
Single	1371 (98.0)
Ever married	28 (2.0)
Nature of camp (*n* = 1399)	
Urban	1125 (80.4)
Rural	274 (19.6)
Fathers’ education (*n* = 1399)	
Less than secondary school	904 (64.6)
Secondary school or higher	366 (26.2)
Not living with father or missing information	129 (9.2)
Mothers’ education (*n* = 1399)	
Less than secondary school	1010 (72.2)
Secondary school or higher	337 (24.1)
Not living with mother or missing information	52 (3.7)
Reported economic status (*n* = 1399)	
Fair	687 (49.1)
Good to very good	526 (37.6)
Very bad to bad	186 (13.3)
Physical activity (*n* = 1397)	
0 day	87 (6.2)
1 day to 4 days	571 (40.9)
5 to 7 days	739 (52.9)
Age at menarche (*n* = 1399)	
12–14 years	1117 (78.9)
< 14 years	106 (7.6)
> 15 years	176 (12.6)
Menstrual cycle (days) (*n* = 1395)	27.3 ± 6.8
Standard (21–45 days)	1278 (91.6)
Frequent (< 1 days)	48 (3.4)
Infrequent (> 45 days)	50 (3.6)
Cannot calculate/do not know	19 (1.4)
Menstrual duration (*n* = 1397)	5.16 ± 1.4
Standard (3–7 days)	1329 (95.1)
Short period (< 3 days)	18 (1.3)
Long period (> 7 days)	50 (3.6)
Heaviness of bleeding (*n* = 1396)	
Never	790 (56.6)
Rarely to sometimes	525 (37.6)
Mostly to always	81 (5.8)

Values are presented as mean standard error or n (%)

### Prevalence and types of PMS symptoms

**Fig. 2:**
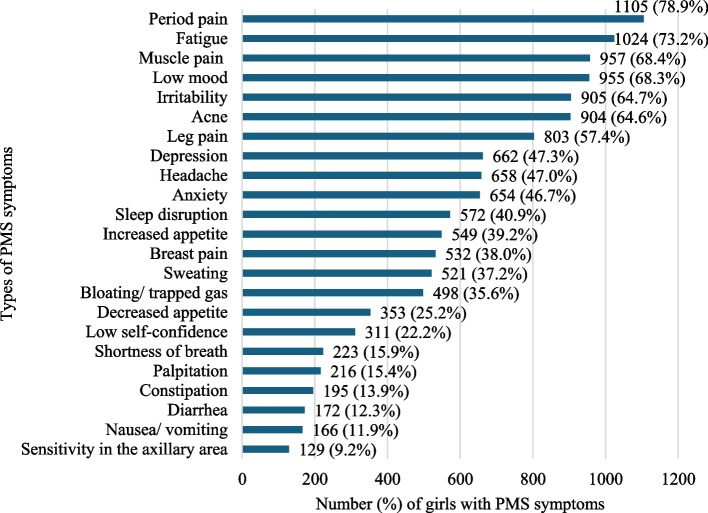
Premenstrual syndrome symptoms among girls (*n* = 1399)

**Fig. 3:**
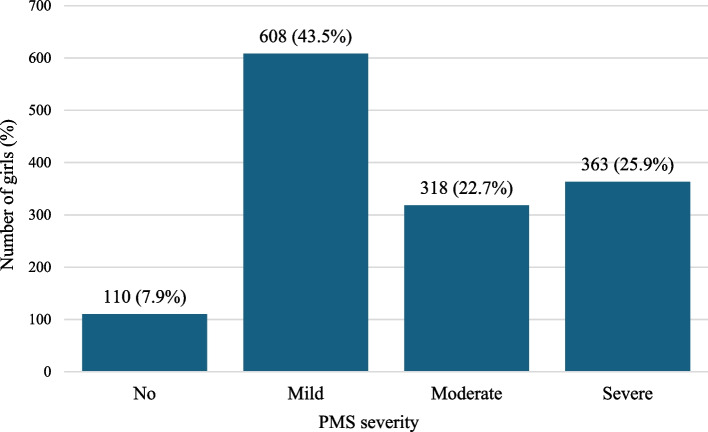
Prevalence of premenstrual syndrome by category severity (*n* = 1399)

The prevalence of PMS among the participating girls is detailed in Fig. 2. The most common symptoms were period pain (78.9%), fatigue (73.2%), and muscle pain (68.4%). Figure 3 shows the prevalence of PMS symptoms at each severity level; 22.7% of the girls had moderate PMS and 25.9% had severe PMS. In total, 92.1% of girls had at least one PMS symptom.

### Prevalence of EPV, human insecurity, and well-being

The prevalence of individual EPV was 47.2%, familial EPV 50.2%, and collective EPV 80.9%, indicating the percentage of participants who had experienced at least one (Fig. 3). The most frequent individual EPV were house searches (44.6%), house occupation (19.7%), and house eviction (9.1%) (Additional File 1). In familial EPV, 42.9% of girls’ families were imprisoned and 20.7% of girls’ families were shot or injured. Additionally, 14.4% of girls’ families were martyred. In the collective EPV, more than two-thirds (71.5%) of the girls were exposed to tear gas, 59.8% to sound bombs, and 40.9% to gunfire. Regarding cumulative EPV, 25.9% of girls reported being exposed to one type of EPV, whereas 63.6% reported being exposed to two or three types, regardless of the type of EPV. Low level of well-being and depression-like symptoms were reported by 22.7% and 11.3% of participants, respectively. High levels of human insecurity were reported by 53.2% of the participants (Table 2).

**Table 2:** Descriptive statistics for girls on exposure to political violence, human insecurity, and well-being (*n* = 1399)

**Variable**	**Category**	**n (%)**
Individual EPV	Never	738 (52.8)
	At least one	661 (47.2)
Familial EPV (*n* = 1395)	Never	695 (49.8)
	At least one	700 (50.2)
Collective EPV	Never	267 (19.1)
	At least one	1132 (80.9)
Cumulative EPV (*n* = 1395)	Never	146 (10.5)
	One type	362 (25.9)
	Two or three types	888 (63.6)
Human insecurity (*n* = 1396)	Low to moderate level	653 (46.8)
	High level	743 (53.2)
Well-being (*n* = 1396)	Good well-being	922 (66.0)
	Low level of well-being	316 (22.7)
	Depression-like symptoms	158 (11.3)

### Association between PMS severity and EPV, human insecurity, and general well-being

We constructed five logistic regression models to carefully examine the form of EPV most strongly associated with PMS severity (Table 3). In Models 1, 2, and 3, women who experienced individual, familial, and collective EPV were more likely to report higher PMS severity than those who did not, and these associations were statistically significant. In Model 4, which included all three forms in the same model, only collective EPV was significantly associated with the severity of PMS (AOR, 1.5; 95% CI, 1.1–2.1). Finally, Model 5 showed a dose–response relationship between EPV and PMS severity. Compared with those who had never experienced EPV, AOR for being exposed to one type or two to three types of EPV were 1.7 (95% CI, 1.1–2.6) and 2.5 (95% CI, 1.6–3.7), respectively. These models were designed to compare the patterns of associations across different types of EPV, rather than to conduct formal multiple hypothesis testing. Although all models shared the same outcome variable, each was based on a distinct theoretical premise. Nevertheless, we acknowledge the increased potential for Type I error due to multiple model comparisons, and we interpreted the findings with caution, prioritizing Model 4, which considers the joint effects of all EPV forms.

**Table 3:** Multivariable analysis for the associations between exposure to political violence and PMS severity (*n* = 1390)

Variables	AOR (95% CI)				
	**Model 1**	**Model 2**	**Model 3**	**Model 4**	**Model 5**
Individual EPV					
Never	Ref			Ref	
At least once	1.4 (1.1–1.7) **			1.2 (0.9–1.5)	
Familial EPV					
Never		Ref		Ref	
At least once		1.3 (1.0–1.6) *		1.2 (0.9–1.5)	
Collective EPV					
Never			Ref	Ref	
At least once			1.6 (1.2–2.2) **	1.5 (1.1–2.1) *	
Cumulative EPV					
Never					Ref
One type					1.7 (1.1–2.6) *
Two or three types					2.5 (1.6–3.7) **

*PMS* Premenstrual syndrome, *AOR* Adjusted odds ratio, *CI* Confidence interval, *EPV* exposure to political violence, *Ref* Reference

*p < 0.05, **p < 0.01

Table 4 shows the multiple binary logistic regression with all covariates for Model 4, in which we included individual, familial, and collective EPV in the same model. This model showed no difference in the results for human insecurity, well-being, and other variables compared to the other regression models. The AOR for PMS severity was higher in girls who felt a high level of human insecurity (AOR, 1.3, 95% CI, 1.0–1.6) than girls who perceived a low to moderate level of human insecurity. A stronger association with PMS severity was observed for girls with low level of well-being (AOR, 1.4; 95% CI, 1.1–1.8) and girls with depression-like symptoms (AOR, 1.9; 95% CI, 1.3–2.7) than for those with good well-being. Girls whose fathers had less than secondary education (AOR, 1.5; 95% CI, 1.1–1.9) were significantly associated with PMS severity. In contrast, the mothers’ education levels were not significantly associated with the severity of PMS. Girls who did not exercise (AOR, 2.5; 95% CI, 1.5–4.1) were more likely to have severe PMS than girls who exercised for 5–7 days. The higher prevalence of PMS severity was observed among girls whose menstrual characteristics do not follow ACOG criteria, such as early age of menarche (AOR, 1.9; 95% CI, 1.2–2.9), frequent menstrual cycles (AOR, 3.3; 95% CI, 1.6–6.6), and frequent heavy bleeding (AOR, 3.0; 95% CI, 1.7–5.1).

**Table 4:** Multivariable logistic regression analysis of the association with PMS severity (model 4) (*n* = 1390)

Variables	AOR (95% CI)	P
Individual EPV		
Never	Ref	
At least once	1.2 (0.9–1.5)	0.100
Familial EPV		
Never	Ref	
At least once	1.2 (0.9–1.5)	0.100
Collective EPV		
Never	Ref	
At least once	1.5 (1.1–2.1)	0.011
Human insecurity		
Low to moderate level	Ref	
High level	1.3 (1.0–1.6)	0.025
Well-being		
Good well-being	Ref	
Low level of well-being	1.4 (1.1–1.8)	0.021
Depression-like symptoms	1.9 (1.3–2.7)	0.002
Mothers’ education level		
Secondary school or beyond	Ref	
Less than secondary	1.1 (0.8–1.4)	0.584
Not living with mother or missing information	0.5 (0.2–1.2)	0.101
Father’s education level		
Secondary school or beyond	Ref	
Less than secondary	1.5 (1.1–1.9)	0.005
Not living with father or missing information	1.3 (0.8–2.2)	0.224
Reported economic status		
Fair	Ref	
Good to very good	0.7 (0.5–0.9)	0.002
Bad to very bad	0.9 (0.6–1.2)	0.416
Frequency of physical activity		
5–7 days	Ref	
1–4 days	1.1 (0.9–1.4)	0.528
0 day	2.5 (1.5–4.1)	< 0.001
Age at menarche		
12–14 years	Ref	
< 12 years	1.9 (1.2–2.9)	0.006
> 15 years	0.9 (0.7–1.3)	0.723
Menstrual cycle		
Normal (21–45 days)	Ref	
Frequent (< 21 days) days	3.3 (1.6–6.6)	0.001
Infrequent (> 45 days)	1.2 (0.6–2.2)	0.586
Menstrual duration		
Standard (3–7 days)	Ref	
Short period (< 3 days)	0.3 (0.1–1.2)	0.088
Long period (> 7 days)	1.2 (0.7–2.3)	0.496
Heaviness bleeding		
Never	Ref	
Rarely to sometimes	1.6 (1.3–2.0)	< 0.001
Mostly to always	3.0 (1.7–5.1)	< 0.001

*PMS* Premenstrual syndrome, *AOR* Adjusted odds ratio, *CI* Confidence interval, *EPV* exposure to political violence, *Ref* Reference

## Discussion

The results revealed a high prevalence of PMS (92.1%) among the study population. Furthermore, PMS severity was positively associated with EPV, particularly collective violence, high levels of human insecurity, and low level of well-being.

Previous studies have reported high prevalence rates of PMS among university and high school students across various countries. Similarly high rates have been reported in the Middle East, including 88% in Jordan [71], 87% in Syria [72], 86.3% in Egypt [73] and, 63% in Lebanon [74]. Among high school students, prevalence rates include 84.9% in Iraq [75], 80.4% in Iran [76].

Differences in prevalence between countries and regions may be due to the different tools and diagnostic criteria used in different studies, as no unified tool is available for PMS assessment at present. Therefore, caution should be exercised while comparing the prevalence and symptoms of PMS [77]. Nevertheless, it is worth noting that the standardized tools reported in the literature for measuring PMS depend on reported symptoms [78, 79]. Furthermore, given that the diagnosis of PMS relies primarily on self-report and recorded symptoms [80], we assumed that our scale would be able to detect PMS in adolescent girls. Most importantly, the high prevalence identified in this study should be seriously considered and addressed.

Available evidence suggests that high stress levels worsen PMS. A Spanish study found that women with moderate to high levels of stress were more likely to experience PMS, emphasizing that psychological and social stress factors contribute to the severity of PMS [78]. Additionally, a study investigating the mental health impact of COVID-19-related stress among Jordanian medical students showed an increase in PMS symptoms such as fatigue, headache, and sleep disturbances [79].

The present study focused on EPV as the primary stressors faced by Palestinians. Our findings suggest that chronic, cumulative exposure to such stressors under prolonged occupation may contribute to the onset of PMS. A high prevalence of EPV was observed among adolescent refugee girls, consistent with prior studies in the West Bank, where up to 85% of adolescents reported some form of exposure to political violence [42, 59].

Long-term conflicts have been reported to negatively affect menstruation. A study in Ukraine found that 65.8% of adolescents reported experiencing irregular menstruation or dysmenorrhea in stressful environments caused by military conflicts [81]. In Lebanon, among women aged 15–45 years who had regular menstrual cycles before the war, the prevalence of menstrual irregularities increased by 36.5% three months after the war [27].

This study also found that collective EPV were associated with the severity of PMS. In contrast, individual and familial EPV were significant only when included individually in the model. This suggests the existence of overlapping effects among individual, familial, and collective EPV. Collective EPV is a widely shared community experience of intentional humiliation alongside many others sharing the same identity [59]. Even if not directly subjected to individual or familial EPV, individuals are exposed to collective EPV as witnesses of political violence and exposure to harsh living conditions under occupation. Collective EPV can be a traumatic experience for communities and a pathway that affects people’s health and well-being more than individual or familial EPV [36, 82].

While individual and familial EPV showed a less significant association with PMS symptoms in this study, their psychological impact warrants further investigation. Prior research on parental detention among Palestinian families has demonstrated substantial mental health consequences for children and adolescents, including increased anxiety, depressive symptoms, and behavioral issues [82–84]. In addition to the trauma of separation, children may experience further distress through punitive measures at checkpoints and verbal abuse during visits to detained family members [83]. Witnessing violence against close relatives can also elicit profound feelings of helplessness and guilt during adolescence, compounding psychological vulnerability [84]. Given these psychological impacts, future research should explore the potential links between individual, familial EPV and PMS severity.

PMS may serve as an early indicator of chronic stress, particularly in environments where mental health issues are stigmatized or underreported [85]. However, PMS itself can also be a sensitive topic, as it is closely associated with reproductive function. Adolescent girls who perceive menstruation as a taboo or uncomfortable subject may hesitate to acknowledge PMS symptoms or seek care, even when such symptoms reflect underlying psychological distress. To utilize PMS as an effective indicator of chronic stress, it is essential to establish supportive environments where adolescent girls have adequate knowledge, feel safe to discuss their symptoms, and are encouraged to seek care.

Human insecurity can be an important health indicator because it offers a more comprehensive understanding of health than traditional measures of morbidity [86]. Human insecurity exists in the form of direct threats, such as shootings, house demolitions, displacement, and imprisonment, as well as indirect threats, such as sieges, barriers, and curfews, that lead to economic restrictions [45]. These threats undermine not only the security of individuals, communities, and Palestinian society, but also access to essential services, economic stability, and freedom of movement. Palestine refugees have experienced multiple episodes of displacement, compounding their detrimental effects and causing deep insecurity and instability [45]. This study revealed that high levels of human insecurity are significantly associated with the severity of PMS. Although no prior studies have directly examined this relationship, previous research in oPt and Syria, where conflicts span decades, has shown that human insecurity is associated with health, particularly mental health, among children and adolescents [28, 87].

In addition to human insecurity, psychological factors such as well-being also appear to play a critical role in the severity of PMS. This study found that low levels of well-being and depression-like symptoms were significantly associated with PMS severity. Well-being often mediates the relationships between stressors and physical and psychological symptoms. A low level of well-being may increase perceived stress, lowering pain tolerance, and amplifying psychological and somatic symptoms [88]. Consistent with this, a study among Palestinian university students in the West Bank reported significant associations between PMS symptoms and both depressive and anxiety symptoms [48, 49]. Furthermore, a Turkish study revealed that the risk of depression contributed to the PMS severity [89]. Interventions aimed at enhancing well-being have been shown to be effective in reducing perceived stress [90]. In addressing PMS and other menstrual health issues, it is therefore important to incorporate a perspective that promotes well-being and is integrated into intervention and support frameworks.

The causal relationship between psychological symptoms and PMS remains difficult to determine, due to the overlapping and interrelated nature of emotional symptoms such as low levels of well-being and depression-like symptoms. This suggests that well-being is both directly and indirectly associated with PMS severity through a bidirectional relationship. Low levels of well-being may intensify PMS, while PMS may further undermine psychological well-being, particularly in contexts of high EPV and elevated human insecurity. Moreover, given that PMS is characterized by complexity and the accumulation of multiple risk factors, EPV may act as a chronic stressor that triggers its onset. It may exert its influence through mediating pathways, including human insecurity, psychological well-being, and menstrual characteristics, which align with the stress response regulated by the hypothalamic–pituitary–adrenal axis and may influence the development of premenstrual disorders including PMS [91–93]. PMS can thus be understood as a biopsychosocial phenomenon shaped not only by biological processes, but also by social, political, cultural, religious, and gendered factors [94].

### Strengths and limitations

This study is the first to identify the prevalence of PMS and its association with EPV, human insecurity, and well-being among refugee girls, by taking into consideration the living conditions under occupation. Our findings highlight the potential impacts of conflict and war on adolescent girls’ health and PMS severity. The large sample size, representing adolescent girls across all Palestine refugee camps in the West Bank, contributes to the validity and reliability of this study. Furthermore, the study was strengthened by the use of validated context-specific tools to assess primary exposures, such as EPV and human insecurity, within a unique population of refugees living under prolonged occupation.

However, this study had some limitations. First, although the PMS has not yet been validated by factor analysis or test–retest reliability, which may limit the empirical validation of its construct validity and temporal stability, the scale was theoretically grounded and developed through a rigorous process, including a literature review, expert input, and the integration of girls’ voices. Further psychometric validation is recommended in future research. Second, this study did not account for individual risk factors such as hormone levels, BMI, smoking, diet, or medication use, which are known to influence PMS. While our focus was on community-level stressors, the absence of adjustment for these potential confounders may have led to an overestimation of the association between EPV and PMS. Third, owing to the cross-sectional design, causal relationships between PMS, EPV, human insecurity, and well-being could not be established. Nevertheless, exploring the association between EPV and PMS in adolescents who have experienced political violence remains essential for understanding broader adolescent health challenges. This study relied on retrospective self-reported data, which may have been subject to recall bias. Participants were asked to report their past experiences of political violence, PMS symptoms, and psychosocial well-being, which may have been influenced by their current emotional state or memory limitations. While this is an inherent limitation of retrospective self-report data, we attempted to mitigate it using clearly defined timeframes and structured questions.

## Conclusions

A high prevalence of PMS was identified among Palestinian adolescents in this study. The severity of PMS was found to be related to EPV, high levels of human insecurity, and low level of well-being. Palestine has been occupied for over 75 years and has been repeatedly subjected to EPV and social and economic constraints across generations, instilling deep feelings of insecurity among the Palestinian population. Long-term occupation and war without a political solution are clear examples of the impact on the health and well-being of adolescent girls, which can manifest in the form of PMS severity. Targeted interventions and policy recommendations are essential in this context.

Several intervention studies have shown that combining psychosocial support with menstrual health education including accurate information about menstruation, stress and anxiety management, and coping skills training can effectively reduce PMS symptoms [95, 96]. Based on these findings, we propose integrating psychosocial support and menstrual health education into schools and community settings such as primary healthcare facilities. These environments provide girls with support from teachers, psychologists, healthcare professionals, and families. We believe that this initiative will help mitigate the psychological impact of EPV and human insecurity, enhance adolescents’ coping abilities, and support the management of menstrual-related symptoms, including PMS. Further research is warranted to explore the specific pathways through which EPV and human insecurity exacerbate PMS severity, as understanding these mechanisms could guide the development of more targeted and context-sensitive interventions.

## Supplementary Information

Additional file 1. Percentage of girls who experienced political violence. This supplementary material shows the proportions and nature of individual, familial, and collective EPV experienced by Palestinian adolescent refugees in the past

## Data Availability

Data are available from the corresponding author upon reasonable request.

## Abbreviations

ACOG: American College of Obstetricians and Gynecologists
EPV: Exposure to political violence
oPt: Occupied Palestinian territory
PMS: Premenstrual syndrome
