# Supplementary material for: Premenstrual syndrome and its association with exposure to political violence, human insecurity, and well-being: a cross-sectional study among Palestinian adolescent refugees
Source: Reprod Health. 2025 Nov 27;22:243. doi: 10.1186/s12978-025-02104-z (PMC12659531; doi:10.1186/s12978-025-02104-z)
Supplement: Supplementary file 1 — Additional file 1. Percentage of girls who experienced political violence. This supplementary material shows the proportions and nature of individual, familial, and collective EPV experienced by Palestinian adolescent refugees in the past [file 12978_2025_2104_MOESM1_ESM.docx]

**Supplment 1: Percentage of girls who experienced political violence.**

Figure 1. The percentage of girls who have experienced individual EPV and its nature (N=1399).

Figure 2. The percentage of girls who have experienced familial EPV and its nature (N=1395).

Figure 3. The percentage of girls who have experienced collective EPV and its nature (N=1399).
